# Microglia Activation and Immunomodulatory Therapies for Retinal Degenerations

**DOI:** 10.3389/fncel.2018.00176

**Published:** 2018-06-21

**Authors:** Khalid Rashid, Anne Wolf, Thomas Langmann

**Affiliations:** ^1^Laboratory for Experimental Immunology of the Eye, Department of Ophthalmology, University of Cologne, Cologne, Germany; ^2^Center for Molecular Medicine Cologne (CMMC), University of Cologne, Cologne, Germany

**Keywords:** retinal degeneration, microglia, TSPO, interferon-beta, photoreceptors

## Abstract

A chronic pro-inflammatory environment is a hallmark of retinal degenerative diseases and neurological disorders that affect vision. Inflammatory responses during retinal pathophysiology are orchestrated by microglial cells which constitute the resident immune cell population. Following activation, microglia cells lose their ramified protrusions, proliferate and rapidly migrate to the damaged areas and resolve tissue damage. However, sustained presence of tissue stress primes microglia to become overreactive and results in the excessive production of pro-inflammatory mediators that favor retinal degenerative changes. Consequently, interventions aimed at overriding microglial pro-inflammatory and pro-oxidative properties may attenuate photoreceptor demise and preserve retinal integrity. We highlight the positive effects of ligands for the translocator protein 18 kDa (TSPO) and the cytokine interferon beta (IFN-β) in modulating microgliosis during retinal pathologies and discuss their plausible mechanisms of action.

## Introduction

With approximately 55 distinct cell types, the retina is an extremely sophisticated and subtle structure (Masland, 2001). It is highly susceptible to a variety of noxious insults including high intensity light, hypoxia, oxidative stress and inherited mutations in retinal genes (Masuda et al., 2017). This necessitates constant surveillance of the retina for the detection of neuropathological signals. Microglia, the immunocompetent resident macrophages, are initially capable to fulfil this function (Langmann, 2007). They are evenly distributed in the plexiform layers and are extensively ramified during homeostatic conditions to enhance surveillance of their microenvironment (Karlstetter et al., 2015). They possess a full assortment of immune surface proteins to sense their environment for “on” and “off” signals (Karlstetter et al., 2015). Such surface proteins include receptors for complement components, cytokines, chemokines and damage-associated molecular patterns (DAMPs). Importantly, neuron–microglia interactions via such surface receptors contribute to the maintenance of retinal homeostasis (Vecino et al., 2016). Examples of reciprocal signals between neurons and microglia that mediate retinal homeostasis include interactions between fractalkine CX_3_CL1—CX_3_CR1, CD200—CD200R and Sialic acids (on neuronal glycocalyx)—Sialic acid-binding immunoglobulin-like lectin-11 (SIGLEC-11; Vecino et al., 2016; Karlstetter et al., 2017). Moreover, microglial cells are continuously required for the maintenance of neuronal synaptic structure and neurotransmission in the adult retina (Wang et al., 2016).

In the event of an insult, such as degeneration due to genetic mutations in the retina, a local immune response involving microglia and the complement system is mounted (Karlstetter et al., 2014b). Microglia cells respond by retracting their filopodia and upregulating cell surface molecules including cytokine and chemokine receptors and major histocompatibility markers (MHC class I and II; Jurgens and Johnson, 2012). In addition, they shift their metabolism towards a Warburg-like effect characterized by increased anaerobic glycolysis with concomitant increase in lactate production (Tannahill et al., 2015). This metabolic event is crucial for the local proliferation that follows shortly. Biosynthetic pathways for nucleotide synthesis, generation of amino acids for protein synthesis and production of lipids for membrane formation, branch out from glycolysis (Orihuela et al., 2016). Subsequently, microglia proliferate and migrate to the damaged layers, releasing a host of pro-inflammatory cytokines, chemokines, reactive oxygen (ROS) and nitrogen species (RNS) as well as neuromodulatory factors to promote the repair of stressed cells (Ferrer-Martín et al., 2015). Moreover, their phagocytic capacity is significantly enhanced to clear debris and cellular corpses at the local surroundings (Kohno et al., 2013).

If the insult is minimal and the stress cue only transient, tissue repair and return to homeostasis is rapidly achieved with minimal alterations in retinal integrity (Chen and Xu, 2015). However, if the insult persists, the initial “constructive” inflammatory response quickly turns destructive and is characterized by overreactive neurotoxic microglia (Karlstetter et al., 2014b). These overreactive resident macrophages release large amounts of pro-inflammatory and cytotoxic factors such as ROS, RNS, TNF-α and IL-1β (Scholz et al., 2015a). Furthermore, overreactive amoeboid microglia cause dysregulation of the complement system by up-regulating the expression of complement activators C3, CFB, C1q and C5AR1 and down-regulating complement inhibitors CFH, CFI, CD46 and CD93 (Madeira et al., 2018). Subsequently, microglial overactivation creates a proinflammatory environment conducive for further recruitment of retinal microglia and exogenous infiltrating monocytes (Zhao et al., 2015). This is clearly demonstrated in studies using bright light to induce retinal degeneration in mice, where microglia recruitment to the outer retina is significantly inhibited in C5aR knockout mice or in mice treated with immunoregulatory agents (Scholz et al., 2015b; Song et al., 2017).

Paracrine factors from reactive accumulating subretinal microglia can then trigger NLRP3 inflammasome activation in the retinal pigment epithelium (RPE; Ambati et al., 2013; Nebel et al., 2017). This is achieved in two stages; first, pro-inflammatory factors such as TNF-α, IL-1α and nitric oxide secreted by reactive microglia would prime RPE cells by activating the NFkB pathway and inducing gene transcription of NLRP3, pro-IL-1β and pro-IL-18; Second, increase in extracellular ATP mediated by the reactive microglia and stressed photoreceptors provides a second hit that causes potassium ions (K^+^) efflux via purogenic P2X7 ATP-gated ion channels resulting in the assembly of NLRP3 inflammasome (Gao et al., 2015). Successful assembly of the inflammasome triggers autocatalytic activation of procaspase-1 into active caspase-1, culminating in the conversion of pro-IL-1β and pro-IL-18 into bioactive peptides (Gao et al., 2015). Inflammasome activation, together with the activation of the complement cascade also shown to be triggered by factors from reactive microglia, induces a chronic inflammatory response and prominent structural alterations in RPE (Madeira et al., 2018). This results in a decline in RPE function as is observed in geographic atrophy (GA), a late stage form of age related macular degeneration (AMD) with concomitant drusen formation (Ambati and Fowler, 2012). Notably, drusen components suppress microglia chemotaxis and promote their retention in the subretinal space, resulting in a vicious cycle of sustained inflammation (Ma et al., 2013). The result is an accumulation of overly reactive microglia in the subretinal space which execute neuronal cell death not only via secretion of neurotoxic factors, but also via indiscriminate phagocytosis of non-apoptotic photoreceptors (Zhao et al., 2015). Furthermore, reactive microglia can induce loss of tight junctions in RPE and enhance their secretion of pro-angiogenic factors, possibly leading to the invasion of abnormal choroidal blood vessels into the retina as seen in wet AMD patients (Ma et al., 2009; Ambati et al., 2013).

There is also accumulating evidence that microglia mediated inflammatory responses are linked to the deleterious effects associated with diabetic retinopathy (DR; Xu and Chen, 2017; Altmann and Schmidt, 2018). Indeed, increased numbers of hypertrophic, amoeboid microglia cells were observed in the outer retina and subretinal space in human DR patients (Zeng et al., 2008). Similarly, hypertrophic, amoeboid microglia localized to the photoreceptor layers of diabetic rats at around 14–16 months where they were probably associated with neuronal loss (Zeng et al., 2000). Hyperglycaemia can induce retinal microglia activity either directly via oxidative stress (Du et al., 2002) or indirectly via effects mediated by stressed retinal cells (Portillo et al., 2017). Oxidative stress in hyperglycemia is driven by a combination of accelerated free radical production by mitochondria and the impairment of antioxidant enzymes regeneration (Nishikawa et al., 2000; Tomlinson and Gardiner, 2008). Hyperglycaemia induced oxidative stress can then cause NF-κB translocation to the nucleus and activate pro-inflammatory pathways in retinal microglia (Du et al., 2002). In addition, CD40 activated Müller cells in high glucose conditions can trigger secretion of TNF-α and IL-1β in microglia and macrophages in a P2X7 receptor dependent manner via release of extracellular ATP (Portillo et al., 2017). Notably, pharmacological blockade or global P2X7 receptor expression diminished the observed upregulation of TNF-α and IL-1β in diabetic mice (Portillo et al., 2017). Moreover, selective P2X7 antagonists prevent high glucose mediated toxicity of cultured human retinal pericytes, indicating that the P2X7 receptor pathway could be an attractive pharmacological target for the management of DR (Platania et al., 2017).

Therefore, inhibiting sustained-microglia mediated inflammatory responses offers a promising therapeutic strategy to attenuate photoreceptor loss and potentially prevent or delay vision deficits. This review article therefore focusses on translocator protein 18 kDa (TSPO) ligands and IFN-β as recent examples that have shown potent immunomodulatory effects in mouse models of light-induced retinal degeneration and laser-induced choroidal neovascularization (CNV). These models recapitulate key biological processes involved in human retinal pathologies such as retinitis pigmentosa (RP) and the exudative form of AMD, respectively.

## Translocator Protein 18 kDa (TSPO) Ligands and Neurosteroids

**Figure 1:**
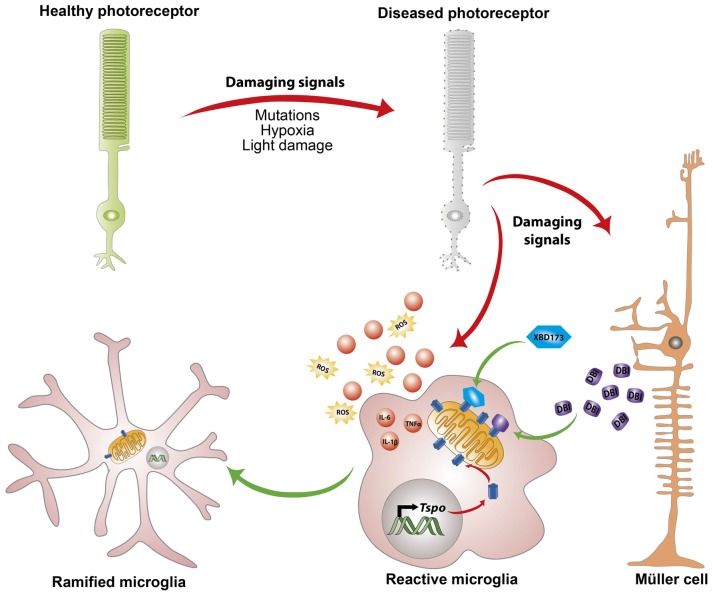
Endogenous and exogenous translocator protein 18 kDa (TSPO) ligands alleviate chronic microglia activation. In response to pathological signals from dying photoreceptors, Müller cells upregulate the expression and secretion of the endogenous TSPO ligand Diazepine binding inhibitor (DBI) protein. Simultaneously, microglia cells upregulate mitochondrial TSPO expression and take up the secreted DBI. Binding of DBI, its cleavage product triakontatetraneuropeptide (TTN) or the synthetic ligand XBD173 limits the magnitude of inflammatory responses and influences transition of microglia towards a ramified neuroprotective phenotype.

Translocator protein 18 kDa (TSPO), previously referred to as the peripheral benzodiazepine receptor (PBR), is a highly conserved 5α-helical transmembrane protein located on the outer mitochondrial membrane (OMM; Rupprecht et al., 2010). TSPO has a high constitutive expression in steroidogenic tissues such as adrenal glands, gonads and placenta, and very low levels in the healthy brain (Batoko et al., 2015). However, during an active neuropathological process, a strong increase in TSPO protein that colocalizes predominantly with activated microglia is observed in the brain and retina (Daugherty et al., 2013). Simultaneously, Müller cells in the retina upregulate the expression and secretion of the endogenous TSPO ligand, Diazepam binding inhibitor (DBI) protein which is in-turn taken up by microglia (Wang et al., 2014). Binding of DBI or its cleavage product triakontatetraneuropeptide (TTN) to TSPO effectively limits the magnitude of microglial inflammatory responses and promotes their return to quiescence (Wang et al., 2014; Figure 1).

Inspired by this endogenous immunomodulatory mechanism, our laboratory tested the ability of a synthetic and highly specific TSPO ligand, XBD173 (AC-5216, emapunil), to influence microglial reactivity in the acute white light-induced retinal degeneration mouse model (Scholz et al., 2015a). Light has been suggested to contribute to the faster onset and progression of human retinal degeneration such as AMD and RP (Heckenlively et al., 1991; Cruickshanks et al., 1993, 2001; Hao et al., 2002; Fletcher et al., 2008). In rodents, exposure to intense visible light results in a significant loss of photoreceptor cells and thinning of the outer nuclear layer (Wenzel et al., 2000; Scholz et al., 2015b). Visible light bleaches the visual pigment rhodopsin, resulting in excessive phototransduction signaling and nuclear translocation of the transcription factor AP-1 (Wenzel et al., 2000; Grimm and Remé, 2013). Induction of the DNA binding activity of AP-1 after light insult ultimately results in photoreceptor apoptosis (Wenzel et al., 2000, 2005). In line with the earlier findings, pharmacological binding of TSPO with XBD173 significantly alleviates microglial pro-inflammatory responses with concomitant inhibition of photoreceptor apoptosis and preservation of retinal structure (Scholz et al., 2015a). However, the central mechanism by which TSPO binding chemicals negatively regulate microglial inflammatory responses remains largely unknown, but likely involves, at least in part, enhanced steroidogenesis (Rupprecht et al., 2010; Midzak et al., 2015).

Indeed, the most studied and well characterized physiological role of TSPO relate to its modulation of steroidogenesis (Midzak et al., 2015). A wealth of evidence implicates TSPO as a translocator of cholesterol from the outer to the inner mitochondrial membrane as a rate limiting step for steroidogenesis (Papadopoulos et al., 2015). Using aminoglutethimide to inhibit the enzymatic conversion of cholesterol to pregnenolone, we have observed that therapeutic effects of the TSPO ligand XBD173 were in-part dependent upon pregnenolone synthesis (Karlstetter et al., 2014a). Similarly, TTN was shown to significantly enhance pregnenolone derived Dehydroepiandrosterone (DHEA) levels with a concomitant attenuation of microglial inflammatory responses (Wang et al., 2014). Outside the retina, pharmacological activation of TSPO with DBI and other synthetic TSPO ligands has also been shown to stimulate steroidogenesis in cell systems and animals (Boujrad et al., 1993; Papadopoulos et al., 2015). Notably, DBI knockdown in Leydig cells significantly suppressed hormone-induced steroidogenesis but not adenylate cyclase nor cholesterol side chain cleavage (P450_SCC_) enzyme activities (Boujrad et al., 1993). Taken together, these findings strongly support the concept that endogenous and exogenous TSPO ligands serve as a pharmacological means to regulate steroidogenesis.

Once produced, steroid hormones rapidly resolve neuroinflammatory process in an autocrine and paracrine fashion (Vasconcelos et al., 2016). They bind and activate their cytoplasmic and nuclear bound receptors, which in-turn blunt the transcription of multiple inflammatory genes (Sever and Glass, 2013). Indeed, the neuroprotective effects of steroid hormones in the retina have been demonstrated in numerous reports. Norgestrel, a synthetic progesterone, was shown to exert powerful neuroprotection against retinal degeneration in an acute light-induced retinal degeneration mouse model and in the *rd*10 mouse model of RP (Doonan and Cotter, 2012). Norgestrel was shown to work, at least in part, via increasing basic fibroblast growth factor (bFGF) levels and by modulating photoreceptor-microglia crosstalk via upregulation of fractalkine-CX3CR1 signaling (Doonan and Cotter, 2012; Roche et al., 2017). Notably, norgestrel was shown in a separate study to work directly on microglia, suppressing expression of pro-inflammatory cytokines, chemokines and nitric oxide and thereby abrogating the associated microglia-driven photoreceptor demise (Roche et al., 2016). Moreover, retinal damage in rats exposed to bright light was significantly ameliorated by 17β-estradiol treatment via enhanced antioxidant genes transcription and ROS inhibition (Zhu et al., 2015). In summary, accumulating evidence highlights TSPO ligands as promising pharmacological agents to modulate microglia activation during retinal degenerative diseases.

## Interferon-Beta Signaling

Interferon-beta (IFN-β) is a type I interferon that possesses strong antiviral and immunomodulatory properties (Stetson and Medzhitov, 2006). IFN-β is an established first-line drug for the treatment of relapsing remitting Multiple Sclerosis (MS), an autoimmune disease that causes demyelination and axon degeneration in the CNS (Limmroth et al., 2011). IFN-β confers neuroprotection in MS by potentiating microglia-mediated phagocytosis of myelin debris with concomitant suppression of neuroinflammatory responses and disease severity (Kocur et al., 2015). Indeed, mice defective in myeloid IFN-β signaling develop an exacerbated disease course and increased lethality in experimental autoimmune encephalomyelitis (Prinz et al., 2008). Based on this evidence, we postulated that IFN-β may have beneficial immunomodulatory effects against chronic inflammatory responses observed in neovascular AMD. To test this hypothesis, we employed the laser-induced CNV mouse model (Lambert et al., 2013). Briefly, laser photocoagulation results in the rupture of Bruch’s membrane, leading to a rapid recruitment of mononuclear phagocytes to the site of damage (Ambati et al., 2013). Enhanced production of pro-inflammatory and angiogenic factors induces the formation and growth of new blood vessels from the choroid into the subretinal space, mimicking features of exudative AMD (Lambert et al., 2013). Using this mouse model, we demonstrated that IFN-β treatment strongly inhibits microgliosis and enhances the morphological transition of microglia towards a neuroprotective ramified phenotype with less Iba-1 signal (Lückoff et al., 2016). IFN-β treatment also resulted in a significant reduction in vascular leakage and neoangiogenesis (Lückoff et al., 2016). In contrast, global (*Ifnar1^−/−^)* as well as microglial specific conditional depletion of IFN-β signaling (*Cx3cr1^CreER^:Ifnar1^fl/fl^*) in mice resulted in exacerbated disease progression (Lückoff et al., 2016). These findings implied that Ifnar1/IFN-β signaling, particularly in retinal microglia, could be targeted to halt disease progression in the laser-CNV model and potentially other degenerative diseases of the retina. Similarly, IFN-β therapeutic effects in the retina have been corroborated in a separate study using a rabbit model, where local administration of IFN-β accelerated the repair of retinal lesions produced by laser photocoagulation (Kimoto et al., 2002).

**Figure 2:**
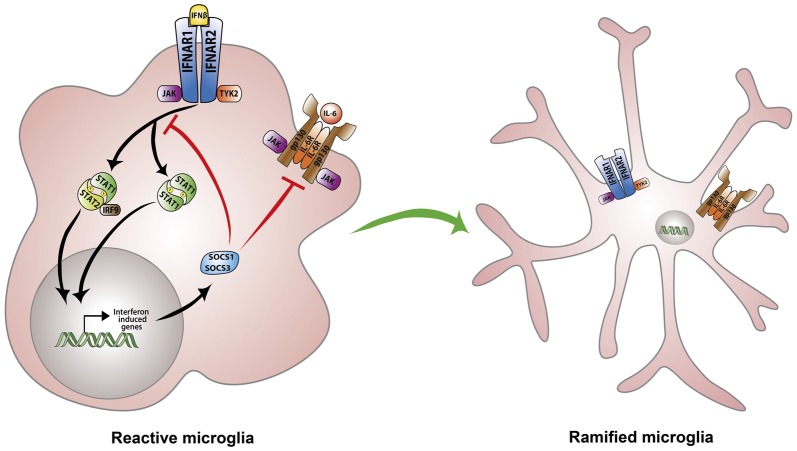
Regulation of microglia responses by IFN-β signaling. IFN-β initiates signaling via binding to the heterodimeric IFNα/β receptor (IFNAR). IFNAR ligation triggers activation of the associated tyrosine kinases JAK1 and TYK2 which in-turn phosphorylate STAT1 and STAT2 transcription factors. STAT1 and STAT2 can also recruit IRF-9 to form a trimolecular complex IFN-stimulated gene factor 3 (ISGF3). STAT homodimers or heterodimers activate the transcription of interferon-stimulated genes (ISGs) including suppressor of cytokine signaling 1 (SOCS1) and SOCS3 as part of a negative feedback loop. SOCS1 and SOCS3 inhibit JAK/STAT and IL-6 signaling thereby preventing excessive cytokine stimulation and dampening microglia activation.

However, despite enormous progress in our knowledge of type-I IFNs signaling, the precise mechanism involved in IFN-β immunomodulatory and anti-angiogenic effects remain poorly understood. This notwithstanding, we discuss in the remainder of this section plausible mechanisms that have been proposed to play a key role in IFN-β negative regulation of neuroinflammatory responses and pathological angiogenesis. It is well known that while IFN-β activates the transcription of interferon responsive genes (ISGs) to establish an antiviral cellular state, it also induces the expression of negative regulators which restrain pro-inflammatory pathways (Ivashkiv and Donlin, 2014). IFN-β induces the transcription of suppressor of cytokine signaling 1 (SOCS1) and SOCS3 as part of the negative feedback circuit aimed at preventing excessive cytokine stimulation (Ivashkiv and Donlin, 2014). SOCS1 and SOCS3 are then recruited to IFNAR receptors where they inhibit JAK/STAT signaling (Yoshimura et al., 2007). Consequently, several reports have highlighted the ability of SOCS1 and SOCS3 to limit the magnitude of inflammatory responses owing to their inhibition of STAT activation (Nakagawa et al., 2002; Whitmarsh et al., 2011). In contrast, SOCS3 deficiency in myeloid cells augments retinal degeneration and accelerates inflammation induced angiogenesis in an experimental autoimmune uveoretinitis murine model (EAU; Chen et al., 2018). Of note, myeloid cell-specific SOCS3-deficient retinas demonstrate higher levels of pro-inflammatory cytokines IL-1β, TNF-α and IFN-γ as well as angiogenic factors including VEGF-A (Chen et al., 2018). Conversely, SOCS1 over-expression in transgenic mice and rats ameliorates disease severity in the EAU model by inhibiting chemokine expression and recruitment of inflammatory cells into the retina (Yu et al., 2011). Moreover, retinal cells overexpressing SOCS1 are protected from staurosporine as well as H_2_O_2_-induced apoptosis (Yu et al., 2011). Overall, compelling evidence implicates SOCS1 and SOCS3 as irreplaceable regulators of type-I IFN signaling and suggest, at least in-part, that IFN-β anti-inflammatory effects are dependent upon these regulatory proteins (Duncan et al., 2017; Figure 2).

IFN-β has also long been known to be a potent activator of the PI3K–AKT–mTOR-signaling axis (Platanias, 2005; Burke et al., 2014). Remarkably, findings from a recent study revealed a striking reduction in Pi3K and Akt mRNA and protein levels in neurons of *Ifn^−/−^* mice when compared to their wildtype counterparts (Liu et al., 2017). The study further reported suppression of active Pi3K/Akt signaling by demonstrating an even more pronounced reduction in phosphorylated (p)Pi3K and pAkt levels in *Ifn^−/−^* neurons compared with IFNβ-competent neurons (Liu et al., 2017). Once activated, the PI3/Akt/mTOR pathway has been shown in several studies to inhibit microglia pro-inflammatory responses (Zhu et al., 2015; Cianciulli et al., 2016). Conversely, pharmacological blockade of PI3K/Akt/mTOR pathway significantly enhances levels of the inflammatory cyclooxygenase-2 (COX-2) and its enzymatic products prostaglandins PGE_2_ and PGD_2_ in primary microglial cultures (de Oliveira et al., 2008, 2012). However, despite mounting evidence, the contribution of this pathway to the immunomodulatory effects of IFN-β on microglia during retinal inflammation and disease warrants further studies.

## Conclusion

There is strong evidence from murine models of experimental retinal pathologies that microglia play a critical role in the development and advancement of retinal degenerative disorders and pathological neoangiogenesis. Therefore, immune based therapies such as TSPO ligands and IFN-β that counter excessive microglia-mediated neuroinflammatory responses and pathological angiogenesis may have an important role in the future clinical management of retinal disorders such as RP and AMD. However, prior to the clinical evaluation of immunomodulatory therapies in retinal diseases, critical questions regarding the exact molecular mechanisms of each individual immunoregulatory compound need to be answered.

## Author Contributions

KR, AW and TL contributed to the concept and writing of the article.

## Conflict of Interest Statement

The authors declare that the research was conducted in the absence of any commercial or financial relationships that could be construed as a potential conflict of interest.
